# Health Security, Quality of Life and Democracy during the COVID-19 Pandemic: Comparative Approach in the EU-27 Countries

**DOI:** 10.3390/ijerph192114436

**Published:** 2022-11-04

**Authors:** Conțiu Tiberiu Șoitu, Silviu-Petru Grecu, Romeo Asiminei

**Affiliations:** 1 Department of Sociology and Social Work, “Alexandru Ioan Cuza” University of Iași, 700506 Iași, Romania; 2 Department of Political Sciences, International Relations and European Studies, “Alexandru Ioan Cuza” University of Iași, 700506 Iași, Romania

**Keywords:** healthcare systems, COVID-19 pandemic, resilience, life satisfaction, democracy, human development, Global Health Security Index

## Abstract

The aim of this paper is to emphasize the role played by the social, economic and political variables in shaping models of sustainable healthcare systems and strategies able to support and improve the quality of life during and after the COVID-19 pandemic. The context of our research is represented by the medical and socioeconomic crises generated by the COVID-19 pandemic. The current pandemic negatively affects healthcare systems, quality of life and the global economy. In this respect, this paper aims to thoroughly scrutinize the effects of the COVID-19 pandemic on the social and healthcare systems of EU countries, to analyze the impact of human development in the field of the Global Health Security Index and to estimate the relation between resilience and quality of life during the COVID-19 pandemic. The research design is quantitative, resorting to the use of both descriptive and inferential statistics, against the background of a long-term comparative approach to the respective situations in the EU-27 countries. Empirical findings are relevant for emphasizing the fact that human development and social progress are predictors for the dynamics of health security measures. Moreover, the quality of the political regime, particularly in the case of full and flawed democracies, is strongly related to a high level of resilience and could influence the perception of quality of life. All of these empirical results could prove valuable for scholars interested in understanding the relationships between democracy, healthcare systems and quality of life, and for political decision makers involved in the effort of reducing the negative effects of COVID-19 in EU-27 countries.

## 1. Introduction

The COVID-19 pandemic is the most complex structural crisis of contemporary global society. It has negatively affected the healthcare systems, global economic dynamics, quality of life and democratic order. The rapid spread of the virus and the high degree of healthcare pressure determined concerted governmental policies related to quarantine and “lockdown” measures. In this respect, “the unprecedented scale and nature of the COVID-19 crisis helps explain why it has generated such an extraordinary surge in economic uncertainty. It remains to be seen which uncertainty measures will prove most useful in explaining economic developments during and after the COVID-19 pandemic” [1]. Moreover, social distancing and “lockdown” measures create issues in the fields of quality of life, social dynamics, economic sectors and healthcare systems [2]. Governmental cooperation in the field of economic and healthcare systems could be seen as an important strategy for limiting the pandemics’ effects and for developing new mechanisms of resilience [3,4,5].

This paper aims to analyze the relation between healthcare, resilience, quality of life, social progress and democratic order in EU-27 countries. This section of the paper presents a brief literature overview related to the impact and the magnitude of the COVID-19 pandemic within the social and political frame. Our research design is quantitative, relying on both descriptive and inferential statistics. We use a comparative approach between the EU-27 countries during 2020–2022 for estimating the relevance of the political dimension in optimizing the level of security and healthcare. 

### 1.1. Healthcare Systems, Resilience and Coordinated Policies during COVID-19 Pandemic: Cross-National Perspectives in EU-27

The COVID-19 pandemic has had a significant impact in the sphere of healthcare systems. Beyond the differences between developed and developing countries, this crisis has generated severe pressure on public health systems. This fact shows the weakness of the global health agenda in a changing world [6,7]. In this respect, we agree with the fact that “we are living in a historic moment, a crisis brought on partially by a virus, SARS-CoV-2, and partially by the de-funding of basic public health systems in the context of longstanding global power differentials” [8]. This pandemic could be seen as a “black swan” both for economic and health systems [9]. Its major impact could be measured through the imbalance observed in the labor market [10,11,12,13,14], the impact of the governmental policies in different economic sectors [15,16,17] and the pressure that characterizes most parts of the national healthcare systems [18,19,20,21].

Resilience is the key concept for understanding the capacity of national healthcare systems to react in such a difficult global situation. In this respect, scholars have emphasized the role played by resilience in shaping an optimal model of reaction during and after the pandemic. For a better understanding of the resilience concept, we argue that it refers to “the capacity of health actors, institutions, and populations to prepare for and effectively respond to crises; maintain core functions when a crisis hits; and, informed by lessons learnt during the crisis, reorganize if conditions require it” [22]. Thus, all governmental interventions and policies tried to recreate “normality” in a context dominated by a severe social and medical imbalance. In the field of resilience, scholars such as Ebi, Semenza and Houston argued that, for decision makers, it is very important to return to “normality” as soon as possible without critical changes in the configuration of the social or political systems [23,24]. Moreover, resilience could be seen as the system’s capacity of being a kind of “shock absorber”. In spite of the fact that, at the theoretical level, there is a lack of consensus regarding the significance of resilience, in practice, we can argue that it reflects the systems’ function of “bouncing back” to normality as quickly as possible in conditions of external or internal threats or other shocks. The origins of the concept are Latin and derive from the word *resilio*, which means “to rebound or bounce back; one definition states that it is the ability to adapt well in the face of adversity or significant stress, even returning stronger afterwards” [25,26]. This function could be related to a low level of entropy for social, economic or healthcare systems. When referring to individuals, resilience consists of a hybrid model, based on personal, biological and environmental factors [27]. When referring to social systems, resilience could be seen as a mix of governmental public policies for reducing the negative impact of an external shock in the field of different social subsystems. In the case of the COVID-19 pandemic, resilience could be seen as a set of governmental interventions in the field of medical, economic and social sectors. These strategies aim to create opportunities for bouncing back to normal life as quickly as possible.

Regarding the resilience issues, scholars developed a complex empirical model for estimating the magnitude of the pandemic shock within the economic, social and medical fields. In this respect, empirical findings suggest that there is a strong linear correlation between the robustness of the sociopolitical systems and the level of resilience. As far as Western countries are concerned, generally speaking, the United Kingdom, the United States and Spain have middle values of the statistical correlation between the robustness of the political systems and the ability to develop resilience mechanisms for limiting the magnitude of the COVID-19 pandemic. In contrast, in Italy, empirical evidence suggests that there is a low statistical correlation between these measures. Only in Scandinavian countries have scholars observed a correlation based on a strong and linear equation model between robustness and resilience. A weekly tracker of GDP growth, mobility behavior, epidemic risk and comprehensive shock index are the main factors that are integrated in the mathematical models for explaining the resilience during and after the COVID-19 pandemic [28]. Recent empirical studies used quantitative models for understanding resilience through nonlinear equations systems, based on two stages: the failure phase and the recovery phase. The failure phase is characterized by shocks and perturbations in social, medical and economic systems, while the recovery phase is characterized by direct interventions for reducing the level of dysfunction and disorder [28].

In accordance with these theoretical perspectives, we present several practices in different geographical areas related to the effort to reduce the negative effects of the spreading of the new coronavirus. For instance, prevention policies, remote learning and working, screening the risk cases, demographic factors, RT-PCR tests and home isolation are the main pillars for health policies and resilience strategies in Arabic countries. These strategies are related to a reduced level of infection and death rates, with 27% in Saudi Arabia. This result could lead to a new strategy for prevention, early control and resilience [29]. The unequal access to health services in Latin American countries such as Colombia and Brazil hindered their pandemic response, in stark contrast to the recommendations of the World Health Organization. The main barriers to accessing care stemmed from delays in testing, challenges in obtaining timely appointments with medical staff, and an insufficient number of available doctors [30]. Cross-national comparisons between various countries in the world reflect the fact that the resilience mechanism is based on several variables, such as health systems, online learning and schools, workforce in healthcare and beyond, proper health policies and international cooperation in the medical research field [31]. We take as examples all of these countries for understanding the dynamics of the EU health strategies during the health crisis. The same pillars of governmental interventions are met in the EU-27 countries. In March 2020, EU-27 national governments decided to introduce lockdown measures. The limitation of social and economic activities and the introduction of online education could be seen as the main pillars for fighting against the spreading of the virus. Concerning European countries, we agree to the fact that the COVID-19 pandemic represents the “biggest challenge since the European communities were created” [32]. This challenge is accompanied by a significant economic depression, material deprivation in vulnerable categories, an increased rate of infection and medical pressure on health systems, high rates of governmental spending and political limitations to civil rights. At the beginning of 2020, health experts tried to identify several possible scenarios related to the spread of the virus and its consequences for the EU-27 countries: “1. short and sporadic chain of transmission; 2. localized sustained transmission; 3. widespread sustained transmission with increasing pressure on the healthcare system; 4. widespread sustained transmission with overburdened healthcare system” [33]. Unfortunately, the data from 2020 and 2021 show that the last two scenarios were incident in most parts of the national healthcare systems in the EU-27 countries. The phases of the European crisis could be analyzed in three steps: full lockdown, gradual opening and open (with some restrictions). In this context, the main challenge for both the European Commission and national governments is to start a strategy based on three “Rs”: repair, reboot and recover [34]. Although Western EU-27 countries are characterized by a high level of democratic order and economic development, in practice, the demographic structures create premises for mitigating the risks for COVID-19 infections and deaths. Belgium, France, Germany, Italy, Luxembourg and the Netherlands are notable for having robust public health systems as well as large elderly populations.

The robust public health systems could not overcome the disproportionate impact that COVID-19 has on the elderly [35]. Related to this observation, scholars specialized in medical sciences and sociology observed that the demographic structure could be seen as an important predictor in the evolution of further epidemic or pandemic times. We can underline the fact that the first year of the COVID-19 pandemic showed the lack of a common response from EU-27 political institutions in managing both the medical and socioeconomic crises [36]. Anderson, McKee and Abel-Smith argued that this pandemic shows the “weaknesses in European response to outbreaks” [37]. For example, since the beginning of March 2020, France, Germany and the Czech Republic have introduced some limitations regarding the export of medical equipment. In spite of the fact that within the institutional design of the European Union exists the Committee or the European Centre for Disease Prevention and Control (ECDC), in practice, we can observe a lot of limitations related to the capability of prevention and intervention.

The empirical results indicate that governmental measures and prevention associated with economic investments could generate opportunities and benefits for the Health Security Index. In this respect, the Global Health Security Index “benchmarks health security in the context of other factors critical to fighting outbreaks, such as political and security risks, the broader strength of the health system, and country adherence to global norms” [38]. This index has the following structure: adherence to international norms, health, response, detection of pathogens, prevention of further epidemics or pandemics and biological risks [38]. Economic and medical resilience could be seen as important predictors of the Global Health Security Index. In this respect, “these issues have become even more important for policy makers and managers due to the financial concerns regarding the continuing economic sustainability and resilience of health systems. The countries’ responses to COVID-19 have been diverse, mainly dependent on the resilience of their health systems, and are still contextual and ongoing issues” [39]. A coherent and common response from EU members could be seen as an important predictor of the magnitude of the pandemic. The main lesson for the EU health systems could be seen in terms of trans-national cooperation and developing trans-national mechanisms of resilience. The situation from Italy, Spain, France and the Eastern European countries could be seen in terms of insufficient resilience, delay in clinical care (for oncological cases, at least), inconsistencies regarding regional cooperation for prevention and regional or national differences in managing the healthcare systems. All of these elements could be regarded as the main threats that are relevant during and after the COVID-19 pandemic. Moreover, we argue that it is very important to have an early warning system and common health policies that could be implemented, depending on the national particularities, in EU member states. The main lesson of the current COVID-19 pandemic could be seen in the following terms: “a proactive and targeted public health response is fundamental for interrupting human-to-human transmission chains and preventing further spread, thereby reducing the intensity of the epidemic” [40]. All of these lessons are derived from the Italian case study. Italy was one of the most affected countries by the negative effects of the new coronavirus. The age of the population, the demographic structure and the significant differences between the north and south could explain the increased rates of infectious diseases and mortality in the northern part of Italy.

The COVID-19 pandemic reflects the fact that “national responses to COVID-19 have varied greatly […] that countries have managed the pandemic differently is expected, but COVID-19 has pushed all health systems to their limits” [41]. In correlation with this fact, we can argue that the rapid and coherent interventions in EU countries have generated the lowest number of cases of infectious diseases and the lowest level of mortality. Delayed governmental interventions are statistically associated with a high rate of mortality and economic costs [42]. In order of importance, a model of best practices in EU countries could integrate the following elements: ensuring the availability of medical equipment for medical staff, common research for discovering treatments and vaccines, supporting the dynamics of the labor market, a common coordination of medical crises and a kind of public–private partnership for pandemic and post-pandemic times [43,44,45,46]. In this context, governmental interventions in the medical field could be based on several actions, such as public procurement of medical equipment (facemasks, ventilators and vaccines), using public funds from both national governments and the European Commission, the increasing of productivity in European industry to cover all medical necessities, and regulations in the field of exports. Besides the medical dimension, EU-27 national governments have introduced “safety economic packages” for supporting national economies. Here, we observe several economic measures, such as the deferral of tax payments, exemption from tax and assistance with the payment of employee wages and preferential loans [43].

### 1.2. Human Development and Life Satisfaction: Challenges and Threats during the COVID-19 Pandemic

The COVID-19 pandemic has represented an urgent sociomedical situation with direct implications in the field of the quality of life for both the young and the elderly [47]. The states’ resilience mechanisms during the COVID-19 pandemic are dependent on the demographic structure, economic activity and social vulnerability. In this respect, the net impact of the pandemic on the sphere of human capital, development and social progress could be measured through Pandemic Risk Exposure Measurement (PREM). Empirical findings suggest that there is a significant global impact of the pandemic in the field of human development, social capital and quality of life during and in post-pandemic times [48]. The net impact of the pandemic, as it is observed in different geographical areas [49], could be analyzed in correlation with human security, human sustainability [50,51,52,53], economic resilience and development, social inequalities and a high level of material deprivation among young and middle-educated citizens [54,55,56,57]. All of these elements could be integrated in the sphere of human development. Education, economy and health could be seen as the main pillars for explaining the umbrella concept of human development.

Human development should be analyzed in accordance with the UN’s assumption of sustainable development (SDG 17). We use the meaning of human development in accordance with the UN definition: “human development is about expanding the richness of human life rather than simply the richness of the economy. It focuses on people and their opportunities and choices” [58]. For measuring human development, the UN uses a composite index named the Human Development Index (HDI) based on a long and healthy life (life expectancy at birth and life expectancy index), knowledge (expected years of schooling, mean years of schooling and education index) and a decent standard of living (GNI per capita and GNI index) [58]. The COVID-19 pandemic has negatively affected the dynamics of the human development index, human capital and sustainable development. Thus, the good cooperation and coordination of health policies could ensure an optimal frame for the decision makers and stakeholders interested in the issues of health and social progress. The World Health Organization (WHO) created a mechanism of coordination among states for reducing the social, medical and economic shock of the current COVID-19 pandemic. Since 2018, the WHO set five phases and responses that should be taken into consideration by governmental actors in epidemic or pandemic conditions: anticipation, early detection, containment, control and mitigation and elimination or eradication [59]. All of these phases are relevant for limiting the spreading of the pathogens and maintaining an optimal level of human development and quality of life. A sustainable model of development could be seen as an important goal, which could be achieved by different countries for reducing the negative socioeconomic impact of the further economic, health, political or social shocks. We agree that “in doing so, the momentum created by the pandemic may lead to a transformation from what currently is regarded as a global threat, to a global opportunity, providing a new impulse leading to the realization of the UN Agenda 2030 as a whole, and of the SDGs in particular” [60]. The COVID-19 pandemic has determined the reassessment of the SDGs, according to geographical, demographic and economic criteria [61].

Empirical studies based on the correlation between the total cases of infections with COVID-19 and the human development index create premises for understanding the dynamics of the virus in sustainable, partially sustainable and non-sustainable countries. More sustainable countries are prone to have a higher level of infections with the new coronavirus and a low or middle rate of mortality. Statistical results confirm that the USA, Spain, Italy, the United Kingdom and Germany are relevant for an increased level of infectious disease and a low or middle rate of deaths/1000 inhabitants [62]. In the academic literature, we have observed that researchers are intrigued by the fact “that many countries of the high HDI group (i.e., values 0.800–0.955), especially in Europe and North America, are those with the worst performance in dealing with COVID-19” [63]. Although there are several statistical associations between the Human Development Index and the positive dynamics of COVID-19 infections, we argue that in a sustainable society, a high level of education and human development could be seen as an important predictor for the fight against further epidemics or pandemic episodes. In this context, we can mention a limited effect related to the lockdown policies. The negative effects of the social isolation and lockdown are reflected in precarious psychological/mental health, macro-economic imbalance and limitations regarding the equal and equitable access to educational resources. Related to the macro-economic imbalances, we take into consideration the high unemployment rates, the high level of inflation and the negative economic growth during 2020 and 2021 [15,16,17]. All of these consequences could be seen as threats for individual wellbeing, social progress and human development [64,65]. The main factors that negatively impacted human development during the pandemic were economic challenges, mental health, gender-based violence, physical health and lifestyle, amount of screen time, misinformation and education [66].

An important finding emphasized in the academic literature shows that the COVID-19 pandemic has had a significant impact in the sphere of education. Although most of the governments decided to transform the academic courses into online remote educational activities, in practice, this situation creates the premise for an increased level of inequality. The asymmetry of resources limited the access to educational systems, an aspect more visible in partially developed or undeveloped countries [65]. In this respect, decision makers have to create new tools and policies for supporting access to the labor market for new graduates [67]. These challenges are relevant for shaping new methods and forms of education in post-pandemic times, using both traditional and new technologies developed and applied in the last two years [68].

Apart from the educational issues, economic and entrepreneurship directions represent other important elements related to human development. In this respect, we can underline the fact that the COVID-19 pandemic has an important impact in the field of business freedom, economic development and the labor market [69]. Economic freedom, entrepreneurship and social development are the main vectors for shaping human development and sustainable societies [70,71,72]. Consequently, the COVID-19 pandemic has generated economic decrease, poverty and social inequalities [73,74,75].

A significant issue related to human development is represented by the satisfaction related to the quality of life during the COVID-19 pandemic. We use the definition of this satisfaction in accordance with the Organization for Economic Cooperation and Development (OECD) perspective. Thus, the OECD presents satisfaction with quality of life in terms of a subjective feeling on a scale between 1 and 10. Thus, life satisfaction “measures how people evaluate their life as a whole rather than their current feeling” [76]. The scale uses 10 as a maximum value, which expresses a high level of satisfaction related to the quality of life. In correlation with this perspective, the European Commission defines quality of life in terms of material living conditions, housing conditions, employment, education, health, social relations, safety, governance and environment [77]. Eurostat uses the same scale as the OECD, from 1 to 10, where 10 represents the highest score associated with a high level of satisfaction with quality of life. In this order, we stress the importance of the social, economic and medical aspects of quality of life. Moreover, an important dimension of quality of life is wellbeing. Although there is a significant risk of affecting wellbeing, in practice, there are no measures for estimating the net impact of the pandemic in this field. Moreover, scholars emphasized the fact that there are coping and resilience mechanisms for both mental and physical wellbeing [78,79]. In this context, empirical studies demonstrate that the lockdown could be seen as a milestone in the analysis of the mental, physical and wellbeing states of individuals. During the lockdown measures, scholars registered the degradation of cognitive processes (memory and cognition), physical energy and happiness among the participants who took part in sociological surveys related to the impact of the pandemic on the social and psychological aspects of life. Body functioning, energy levels, social activities, social isolation and personal factors are seen as the main predictors for the positive relation between wellbeing and quality of life during and post lockdown measures [80,81]. Moreover, other important quantitative studies show that there is a linear correlation between education, satisfaction with quality of life and social media exposure [82]. Regional studies from Italy, Spain and Asian countries show that the COVID-19 pandemic generated both psychological and physical distress. Moreover, the rate of distress was higher among women and adolescents. Statistical results from different geographical areas and political systems confirm that high percentages of psychological distress and negative satisfaction associated with wellbeing and quality of life depend on gender, education and age [83,84,85,86,87,88,89].

The satisfaction with quality of life and the level of wellbeing is strongly associated with psychological anxiety during and post lockdown measures. Social distancing, isolation and the magnitude of the COVID-19 infections created the premise for an increased level of anxiety within different professional categories. Medical staff are more exposed to this phenomenon. In this respect, we argue that “many suffer from uncertainty, fear of infection, moral distress and grief, often experienced alone. There is increasing concern about coping with the resulting anxiety, as well as with its long-term individual and collective impacts” [90]. Traumatic stress, burnout and depression are the main threats for mental and physical health during and after pandemic periods. All of these elements are incident in both medical staff and the general population in most parts of the world. When analyzing the job-related causes of stress for medical staff, sociologists observed that the risk for mental distress and low satisfaction with quality of life could be associated with several variables and occupations, such as mental health professionals, emergency medicine workers, a history of mental health issues, the perceived risk of contracting coronavirus, emotional functioning and age [91,92]. Besides medical staff, teachers (from all educational levels: primary, elementary, college and university) were also affected by the spread of the new coronavirus. Remote working negatively affected educational staff and the performance of students during lockdown measures. A high number of hours worked at home during the pandemic and the negative balance between work and family life are the major elements that could be related to teachers’ quality of life and wellbeing [93,94,95].

The cost–benefit analysis of lockdown policies reflects the fact that there are serious implications of the social distancing and isolation in the field of human development, including satisfaction with quality of life and wellbeing. To highlight this observation, we agree with the scholars’ assumptions and analysis related to the impact of the governmental policies for limiting the spread of the new coronavirus in the sphere of the social, psychological and economic dimensions: “this suggests that the costs of continuing severe restrictions are so great relative to likely benefits in lives saved that a rapid easing in restrictions is now warranted” [96]. Beyond this aspect, we could emphasize the fact that the COVID-19 pandemic related to an increased rate of mortality in most parts of the globe. The empirical studies developed since the end of the 2020 confirm that there are significant variations in the mortality rate generated by both the COVID-19 infectious diseases and the limited access of individuals to healthcare systems. The impact of the current pandemic could be more serious from a long-term perspective. In this respect, “although COVID-19 might be seen as a transient shock to life expectancy, the evidence of potential long-term morbidity due to long COVID and impacts of delayed care for other illnesses” [97]. In correlation with the health and global economic impact [98], we can stress several lessons developed by researchers during COVID-19. Daniel T.L. Shek identified twelve issues relevant during the COVID-19 pandemic: digital divide, health inequalities, gender inequality, poverty, family wellbeing, quality of life, economic growth, consumption versus environmental protection, individual versus collective rights, international cooperation versus international competition, prevention of negative wellbeing, and positive quality of life during the COVID-19 pandemic [99].

Regarding life satisfaction, we are interested in emphasizing the importance of the social and political factors. For the social dimension, we use the Social Progress Index as quantitative variable. The Social Progress Index is a statistical measure published since 2013, which reflects the “country’s wellbeing that is independent of economic indicators” [100]. Social progress is defined as “the capacity of a society to meet the basic human needs of its citizens, establish the building blocks that allow citizens and communities to enhance and sustain the quality of their lives and create conditions for all individuals to reach their full potential” [100]. This index is based on three pillars: basic human needs, foundations of wellbeing and opportunity. All of these components are measured on a scale between 0 and 100. The maximum value of the scale (100) shows the highest level of social progress. Related to the political dimension, we take into account the Index of Democracy. The Index of Democracy measures the quality of the political regimes in 167 world countries. This index “covers almost the entire population of the world and the vast majority of the world’s states (microstates are excluded). The Democracy Index is based on five categories: electoral process and pluralism, functioning of government, political participation, political culture, and civil liberties” [101]. Based on the values related to political participation, political pluralism, participative political culture and the functioning of government, “each country is classified as one of four types of regime: full democracy, flawed democracy, hybrid regime or authoritarian regime” [101]. The data are measured at a ratio level between 0 and 10, with the following labels: values between 0 and 4 are associated with authoritarian regimes, values between 4.01 and 6.00 are associated with hybrid regimes, values between 6.01 and 8.00 are associated with flawed democracies and values between 8.01 and 10 are associated with full democracies.

All of these issues could be seen as important directions that should be analyzed for explaining the complex and dynamic relation based on human development, quality of life, wellbeing and political implications for diminishing the negative impact of the new coronavirus in a global world.

## 2. Research Methodology

Related to the theoretical perspectives underlined in the literature review section, this article presents the relation between healthcare systems, human development, quality of life and democracy using a quantitative design from a long-term statistical perspective. Our paper aims to support the idea that full and flawed democracies prove to be more compatible with a robust healthcare system and social progress index. Democratic regimes could be more prone to develop resilience strategies related to pandemic times. In this respect, we argue that the quality of the political regime could be an important predictor of the evolution of the social, medical and economic indicators during the COVID-19 pandemic. This section of the paper introduces the theoretical research design, data and statistical procedures, research methodology and quantitative tools.

### 2.1. Theoretical Design, Research Objectives and Hypotheses

For a better understanding of healthcare, quality of life and human development correlations, we start the current analytical approach with several research questions: 1. What is the magnitude of the impact of the COVID-19 pandemic in the sphere of the Health Security Index? 2. How could the quality of the political regime influence the dynamics of the healthcare policies in the EU-27? 3. How could the political regime influence quality of life? 4. What is the relation between social progress and human development in the sphere of social security? 5. How could the resilience of political systems influence the quality of life in EU countries during the COVID-19 pandemic?

From a theoretical point of view, this paper aims to create a comprehensive model for a better understanding of the political impact of democratic regimes in the sphere of human development and quality of life during pandemic periods. In accordance with these premises, the research objectives of the study are:

Objective_1_ (O_1_): To perform a thorough analysis of the COVID-19 pandemic in the social and health systems of EU countries.

O_2_: To analyze the impact of the quality of democracy in the field of the Health Security Index.

O_3_: To estimate the impact of human development and social progress in the sphere of the Health Security Index during the COVID-19 pandemic.

O_4_: To determine the relation between the quality of democracy and satisfaction related to quality of life.

O_5_: To estimate the impact of resilience for reducing the negative impact of further social and health crises.

This study aims to test several hypotheses, as follows:

**Hypothesis 1 (H_1_).**  *Democratic systems are more prone to be resilient during the COVID-19 pandemic.*

**Hypothesis 2 (H_2_).**  *Human development and social progress are relevant factors in explaining an increased Health Security Index level.*

**Hypothesis 3 (H_3_).**  *Resilient political systems are more prone to be associated with a high level of satisfaction related to life satisfaction.*

**Hypothesis 4 (H_4_).**  *Democratic countries are more prone to be correlated with a high level of life satisfaction.*

### 2.2. Data, Methods and Statistical Procedures

In order to create the nexus between the theoretical approach and the empirical findings, we propose a quantitative design for exploring and explaining the dynamics of the healthcare systems, human development and quality of life in correlation with the quality of national democracy during the COVID-19 pandemic. As research methods, we use the comparative case studies between countries from different geographical areas with full or flawed democracies. The data were collected from secondary sources as indicators constructed by international think-thanks. The quality of the national democracies is measured by the Economist Intelligence Unit (EIU) through the Index of Democracy. We collected data regarding the dynamics of the Human Development Index from the official site of the United Nations Development Programme (UNDP). The official site of the EU is used in association with the OECD measures of the satisfaction related to quality of life and life expectancy during 2020–2022. The economic indicators that refer to the dynamics of economic growth and GDP/capita are measured by the World Bank. Besides these measures of socioeconomic data, we use the Resilience Index developed by FM Global and the social progress index measured by Social Progress Imperative, a global NGO that provides social, environmental and health data both for decision makers and citizens, as research variables. The research variable related to the healthcare system is extracted from the Global Health Security Index (GHSI). Other healthcare and medical variables associated with COVID-19 infections, rate of mortality, vaccination rate or demographic variables are extracted from the measurement developed by Johns Hopkins Resource Center. In Table 1, we present the research variables, units of measurement and sources of data.

At the statistical level, this study uses both elements of descriptive and inferential statistics. Related to the descriptive statistics, we use central tendency measures (mean, median, mode and percentiles), dispersion (variance, standard deviance and range) and measures for the statistical distribution for the main research variables (Pearson’s moment of the skewness coefficient for asymmetry and the kurtosis of the statistical distribution). All of these statistical measures of the central tendency are useful for creating an adequate image and radiography of the dynamics of the healthcare systems and social indicators during the COVID-19 pandemic. As elements of inferential statistics, we use the theory of probabilities, linear and polynomial equations of regressions and several elements of differential calculus. Moreover, we aim to identify the relations between the dependent variables of healthcare systems, quality of life and human development and independent factors such as political, economic or medical indicators. Our analytical model is based on a multi-linear equation of regression, solved using both stepwise and forward methods. In accordance with the quantitative approach, we propose the following design [105]:

Let be X_i_ and Y_i_, where Y_i_ = dependent variable and X_i_ = independent variables,

(1) $$\mathrm{If}\;X_{i}\;\mathrm{has}\;\mathrm{values}\;X_{i}=\{X_{0},\;X_{1},\;\ldots ,\;X_{n}\},\;X\in R$$

(2) $$Y_{i}\;\mathrm{has}\;\mathrm{values}\;Y_{i}=\{Y_{0},\;Y_{1},\;\ldots ,\;Y_{n}\},\;Y\;\in R\;$$

The multiple linear equation of regression is:

(3) $$Y=\alpha +\beta_{1}x_{1}+\beta_{2}x_{2}+\cdots +\beta_{n}x_{n}+u_{ij}$$

where α = intercept, $\beta_{1,n}$ = slope and coefficients of regression and $u_{ij}$ = residuals.

**Figure 1:**
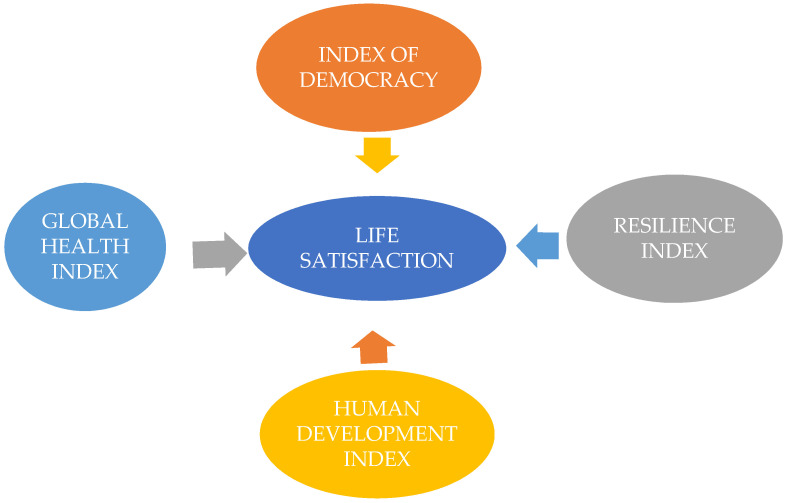
Correlation between research concepts.

Regarding the methodological guidelines, we aim to underline that this study uses comparative case studies between 27 political systems of the European Union. The paper presents the dynamics of the medical, social and economic indicators in correlation with political variables in the EU-27 during the COVID-19 pandemic. In this context, we use long-term statistical data from 2020–2022. The data are relevant from March 2020 to September 2022, covering the whole period characterized by the COVID-19 pandemic. In accordance with our research design, we propose the graphical model from the Figure 1 for stressing the correlations between research constructs:

The following section presents and explains the main empirical findings related to the impact of the COVID-19 pandemic in the field of healthcare systems, human development, quality of life, resilience, social progress index and their correlation with the quality of national democracy. Statistical results are relevant at the *p* < 0.05 level of likelihood.

## 3. Results

This section presents the main empirical findings associated with the research objectives of the study. We use three subsections for emphasizing the COVID-19 situation in the EU-27 during 2020–2022, the relation between healthcare systems and human development and several correlations between resilience, quality of life and democracy. We aim to emphasize that democratic regimes are more prone to develop political strategies for optimizing quality of life and social progress during pandemic periods. Moreover, geographical disparities are relevant for explaining the dynamics of medical, social and economic variables during and post pandemic times. Statistical estimations show that the EU-27 has a middle value on the Global Health Security Index, which reflects a limited capability to prevent and respond to different health threats such as pandemics or epidemics. This measure could be seen as an important predictor of the dynamics of healthcare systems. It could be integrated into a kind of “early warning unit” for detecting further threats and challenges in association with human development, quality of life and social progress.

### 3.1. Impact of COVID-19 Pandemic in EU-27 Countries

As we have already mentioned in the first part of the paper, COVID-19 has had important impacts on the social, economic and medical aspects of life both at the global and local levels. In this respect, the EU-27 is characterized by several features that could create the image of partially functioning health systems and a lack of coordination in the sphere of health policies. Regarding the GHS, we support the idea that the EU-27 is characterized by a partially reactive and preventive mechanism for current and future epidemics and pandemics. Table 2 presents the summary statistics for some relevant variables from the social, healthcare and political dimensions.

In accordance with the results from Table 2, the values of GHS reflect the average of the index of 57.32 with σ = 7.92. The confidence level for GHS with *p* = 0.05 has values between 54.25 and 60.40. This measure reflects the fact that in the EU-27, there are several limitations related to the state capacity of coordinating the healthcare policies when biological or epidemiological threats are imminent for the whole society. An important measure of the quality of the political system is represented by the Democracy Index. In this context, the average of the Democracy Index is 7.80 with σ = 0.85, and (1 − *α*) = [7.47–8.13], *p* = 0.01, a fact that suggests that most parts of the EU-27 political systems could be placed in the sphere of flawed democracies. The Human Development Index has a high average (0.898) with very small values for variance and standard deviation (0.03), a fact that indicates that most parts of the EU-27 could be integrated in the sphere of developed and sustainable countries. At the same time, high values are incident for the Resilience Index and social progress index (>80.00). Although the average of the Resilience Index is very high (80.66), we estimate a high value of standard deviation (12.07), which reflects a middle tendency to partial resilient systems. In this respect, we estimate that the confidence level (1 − *α*) = [75.98–85.33] with *p* = 0.05. The social progress index has the highest values of the statistical series (86.42) with a low level of variance and deviation (3.70), a fact that could indicate that EU-27 countries could be seen as interesting examples in terms of social progress and development. An important variable is represented by the satisfaction with quality of life. In this respect, both OECD and EU measures reflect a middle value on the scale between 1 and 10 (6.23 with σ = 1.90). Thus, we can estimate that EU-27 citizens have partial satisfaction related to the quality of their lives and mental or physical wellbeing. As we pointed out in the first section, quality of life and wellbeing (both mental and physical) have known negative variations during the COVID-19 pandemic.

**Figure 2:**
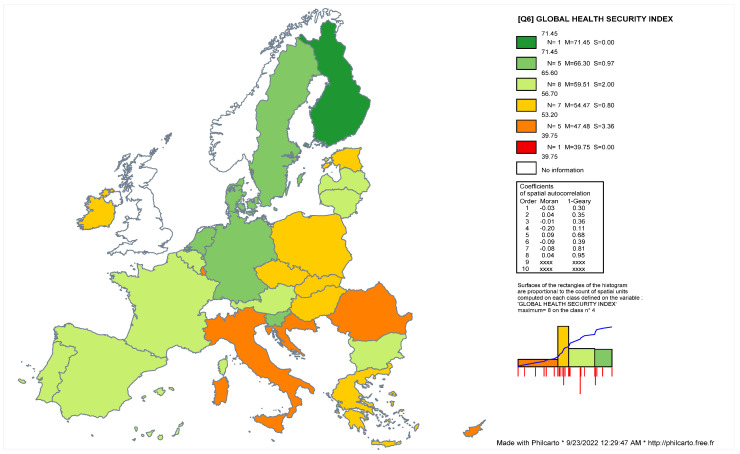
Values of the Global Health Security Index in the EU-27. Average for the period 2020–2021. Source of data: Global Health Security, 2021: https://www.ghsindex.org/about/ (accessed on 10 July 2022). Values > 65.00 are relevant for an increased Global Health Security Index level. Values < 53.00 are relevant for low values associated with the Global Health Security Index.

The Figure 2 shows the geographical distribution of the Global Health Security Index (GHS) across the EU-27 countries. Quantitative measures reflect that the Moran Index (−0.03) is associated with a random distribution of the phenomenon and the Geary Index (0.30) shows that there is a small positive local autocorrelation between the units of analysis in the distribution of the health security indicators. In the first quartile (Q_1_ = 53.33) are integrated countries from the eastern and southern part of the EU-27. Most of the eastern countries are relevant for their communist past and low scores associated with the Democracy Index. In this category are countries such as Romania (GHS = 45.6), Croatia (GHS = 49.3) and Slovakia (GHS = 53.2). Alongside the post-communist countries in this cluster are incident values for Cyprus (GHS = 42.1) and Italy (GHS = 51.9). The statistical values associated with the average of the index are relevant for countries such as Poland (GHS = 55), Ireland (GHS = 55.2), Estonia (GHS = 55.55), Austria (GHS = 57.15), Belgium (GHS = 60.6), Bulgaria (GHS = 60.65) and France (GHS = 62.25). In contrast, the highest values associated with healthcare measures are found for Scandinavia and the northern part of the EU-27. In this respect, Finland can be seen as an example of good practices related to healthcare, having the highest values of detection, prevention and healthcare (GHS = 71.45). In this category, based on the Q_3_ = 64.76, we can include several northern European countries such as Denmark (GHS = 65.85), Sweden (GHS = 65.65), the Netherlands (GHS = 66.2) and Germany (GHS = 65.6). Starting from these statistical descriptions, we stress the fact that there is only a middle positive association between the quality of healthcare systems and the level of democracy in full democratic political regimes. A quantitative estimation indicates that there is a quadratic equation of regression with middle positive values that underline the association between GHS and the quality of national democracy (R^2^ = 0.51, *p* < 0.01). In the case of flawed democracies, the statistical results are not relevant for understanding the correlation between the quality of the political regimes and the level of development in the field of health security. The empirical findings suggest that there is a nonlinear association between political regimes and healthcare measures. Full democracies are more prone to develop sustainable healthcare systems able to detect and respond quickly to different forms of biological or epidemiological threats.

Related to the impact of the GHS in the field of confirmed COVID-19 cases, we estimate a very weak statistical correlation among all of the EU-27 countries with R^2^ = 0.11, *p* < 0.05. If we split the data into developed countries and full democracies only (Scandinavia and the northern part of the EU-27), we estimate a cubic equation of regression with a negative association between the score of the GHS and the confirmed cases of COVID-19 infections. In this respect, we estimate that this negative relation could signify the fact that an increased level of detection, prevention and treatment for pathogens could ameliorate the number of confirmed cases and could decrease the medical pressure on the national public healthcare systems (R = −0.71, R^2^ = 0.51, *p* < 0.05). In this context there is no significant statistical correlation between the quality of the political regime and the dynamics of the COVID-19 confirmed cases.

### 3.2. Health Security, Social Progress and Human Development: Challenges and Interactions during COVID-19 Pandemic

In accordance with the theoretical perspectives, we show that the Global Health Security Index (GHSI) could be seen as an important measure of the pandemic effects in the sphere of medical and social sectors. The statistical results indicate that GHS has middle values in the EU-27, except several countries from Scandinavia. In this respect, we are interested in analyzing the statistical correlations between GHS and other social, economic and political variables for observing several predictors that could be useful for increasing the GHSI level in the EU-27 countries. These results could be seen as important findings for both decision makers from national governments or international organizations and scholars from medical and social science fields interested in measuring the impact of further epidemics or pandemics on social life. In this respect, we observe that human development and social progress could be seen as important sociological variables that could explain the dynamics of the GHS during pandemic times. As we pointed out in the theoretical section, human development and quality of life are strongly related to the dynamics of the pandemic and to the governmental policies for reducing the spread of the virus.

Quantitative estimations suggest that the social progress index could be an important predictor with β = 0.504, *p* = 0.006 for GHS as dependent variable. In addition, the model respects the collinearity diagnostics criteria with a tolerance = 1.00 and VIF = 1.00. Thus, the social progress index captures the UN prospects for SDG 17 in a composite measure related to the level of sustainable development in different types of societies. In this respect, the social progress index, as a measure of sustainable development, could predict the evolution of the healthcare dimension in terms of sustainable systems.

**Figure 3:**
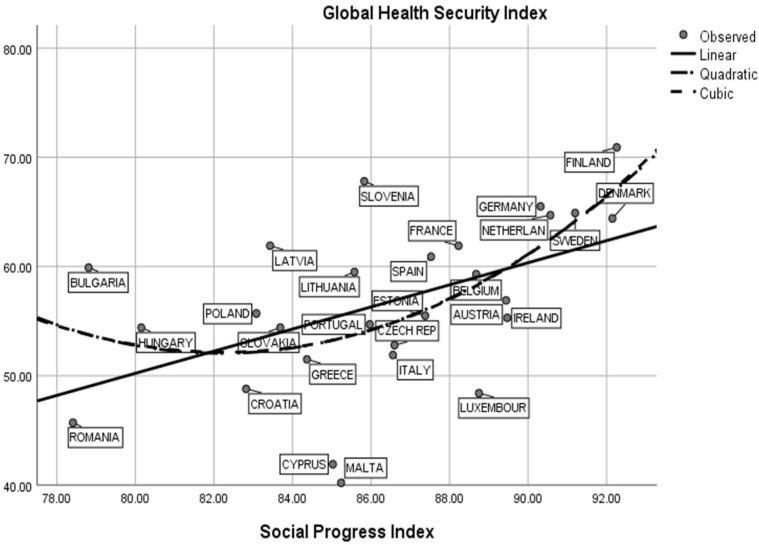
Correlation between the Social Progress Index and the Global Health Security Index. Author’s quantitative determination based on statistical data presented in Section 2 (Research Methods).

**Figure 4:**
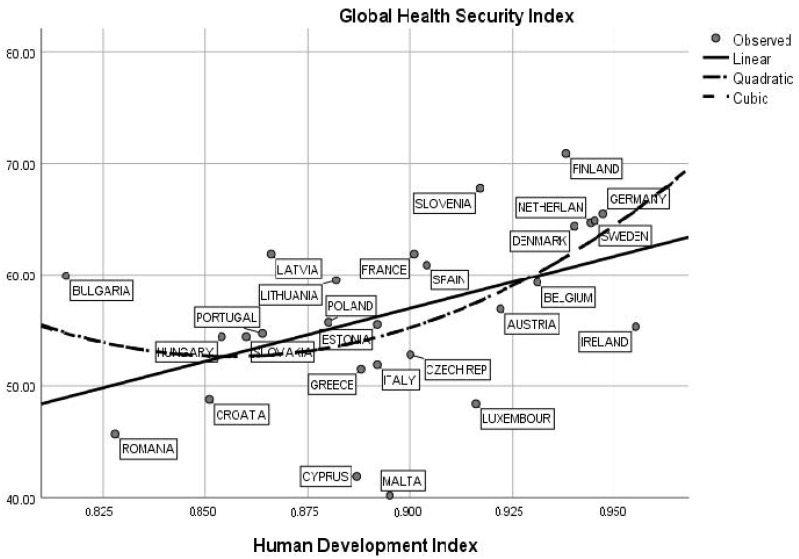
Correlation between Human Development Index and Global Health Security Index. Author’s quantitative determination based on statistical data presented in Section 2 (Research Methods).

Figure 3 shows that there is a moderate correlation between social progress and Health Security Index in EU-27 during 2020–2022. In this respect, the graph suggests that Scandinavian and northern European countries are relevant cases for an increased relationship between the social progress index and a high Health Security Index level. Thus, in countries such as Finland, Denmark, Sweden, the Netherlands and Germany, we estimate a strong positive statistical correlation between the Health Security Index and the level of sustainable development measured through social progress. For these countries, we estimate that R^2^ = 0.70, *p* < 0.001. In contrast, eastern European countries such as Romania, Bulgaria, Poland, Slovakia and Croatia are characterized by middle values related to the social progress index and low scores for health indicators. In these cases, we estimate a very low statistical association between research variables with R^2^ < 0.10, *p* < 0.01. The statistical results confirm that in countries characterized by a high average of the social progress index, there are high values associated with medical indicators as the Health Security Index. Sustainable countries are more prone to and interested in developing good practices for detection, prevention and treatment in epidemic or pandemic periods. Figure 4 presents a similar statistical result in the sphere of human development and Health Security Index interactions. This result emphasizes the fact that Scandinavian and northern European countries are characterized by an increased human development index level (HDI > 0.90) and above-average values related to the Health Security Index (GHS > 65.0). In accordance with these assumptions, we stress the idea that sustainable and developed societies are relevant for creating mechanisms for fighting against further biological or epidemiological threats. An increased level of education related to economic performance, as part of human development, could be an optimal strategy for developing research programs and “early warning systems” in the field of pathogen detection.

The economic, educational and social dimensions of social progress and human development could be explained through an increased Democracy Index level and economic growth. In this respect, we use the multiple linear equation of regression as analytical model for identifying several significant predictors for the Health Security Index. The statistical results confirm, with an R^2^ = 0. 891, *p* = 0.01 and Durbin–Watson = 2.05, that there are two significant predictors for the Health Security Index that are specific to full democratic states only. In this context, we argue that only in full democracies can the quality of political and economic systems be seen as an important predictor of the dynamics of health security indicators.

Table 3 indicates that there are two statistical models for explaining the dynamics of the Health Security Index across EU-27 countries. Thus, we opt for the second model of multiple linear equations based on the following relation:

(4) GSH=15.85+0.547×ID – 0.955×GDP

**Figure 5:**
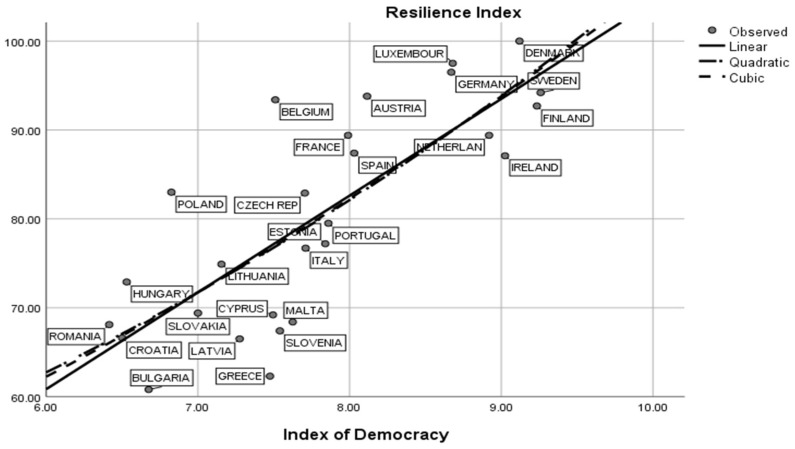
Correlation between Democracy Index and Resilience Index. Author’s quantitative determination based on statistical data presented in Section 2 (Research Methods).

The model has no collinearity diagnostics with tolerance = 0.905 and VIF = 1.104, and T = 4.45, *p* = 0.02 for the democracy index and T = −7.73, *p* < 0.01 for GDP/capita. This model underlines the fact that for full democracies, the healthcare dimension could be explained by a mix of political and economic factors. In this respect, the quality of democracy is partially positively correlated with an increased Health Security Index value. Democratic countries are more prone to invest in and sustain the health sector through medical research, increased capacity of detection and prevention related to the different pathogens, including SARS-CoV-2, and new treatment mechanisms for reducing mortality and medical risk in the general population. Related to this fact, we can observe that democratic systems are more resilient in medical, economic or social crises than other types of political regimes (flawed democracies or hybrid political regimes). In this respect, Figure 5 expresses the linear correlation between democracy and the Resilience Index, with R^2^ = 0.610, *p* < 0.01.

In Figure 5, high democracy index values are strongly correlated with high levels of resilience in countries such as Denmark, Sweden, Germany, Finland, Luxembourg, the Netherlands and Austria. Generally speaking, Scandinavian countries are more relevant for understanding the association between resilience as a rebound mechanism and democracy as political regime based on civil liberties, political rights, political participation and deliberation and free and fair elections. In this respect we observe that in Scandinavian countries, the relationship between the Health Security Index and resilience could be observed in correlation with the total confirmed cases of COVID-19 infections. For example, Finland registered a proportion of 23.14% and Sweden a total of 25.22% confirmed cases from the total population. The average of confirmed COVID-19 cases in Scandinavia is 33.6% of the total population and the average in the EU-27 is estimated at 38%. We observe that the more resilient, developed and democratic countries from northern EU-27 countries registered a decreased level of confirmed COVID-19 cases in comparison with western and eastern parts of Europe. In contrast, low values related to resilience and democracy are incident in post-communist Europe: Romania, Bulgaria, Croatia, Hungary and Slovakia. In this framework we can also integrate other countries such as Greece and the Baltic states. In these political systems, there are middle values associated with democracy (flawed democracy) and low values associated with the Resilience Index.

In synthesis, this part of the paper shows that the health security dimension is correlated with both economic and political factors. Countries characterized by high levels of human development, social progress and economic growth are more prone to develop qualitative healthcare systems for responding and reacting quickly to epidemiological threats. Moreover, the quality of the political regime could be an important empirical parameter that suggests that medical factors are heavily dependent on the level of openness and stability of the political system. Full democratic countries, such as those from the northern part of Europe, Scandinavian countries and Ireland represent several examples that could confirm that a high level of democracy is strongly related to resilience and a better system capacity for managing difficult cases and situations of COVID-19 infections.

### 3.3. Quality of Life, Democracy and Resilience during Pandemic Times

Democracy and resilience could be seen as important key concepts and variables for researchers interested in the field of health policies during and following the COVID-19 pandemic. Quality of life is an important parameter related to human development and wellbeing during and after the pandemic. Beyond the economic and medical aspects, sociological and psychological dimensions of social crisis could be seen as important consequences of the current pandemic.

The statistical model used for explaining the dynamics of the satisfaction related to quality of life is a multiple linear equation of regression with R^2^ = 0.752, *p* < 0. 01. Moreover, we estimate that the normal values for the autocorrelation coefficient of residuals of the Durbin–Watson are ≈ 2.1. The model uses all of the variables into a multiple linear equation of regression, solved using both stepwise and forward methods. Measures of the collinearity diagnostics are tolerance = 0.466 and VIF = 2.146.

Table 4 shows that in model 2, there are two significant predictors related to the satisfaction of the quality of life with the following relation:

(5) QL=15.85+0.521×ID+0.410×RI

In accordance with this result, we confirm that political variables such as the quality of democracy and the capacity of the system to rebound during crises are key variables for understanding the dynamics of human development, quality of life and wellbeing. In this respect, we can observe that in full democratic countries (*ID* > 8.00) there are high levels of satisfaction related to the quality of life during 2020–2022.

**Figure 6:**
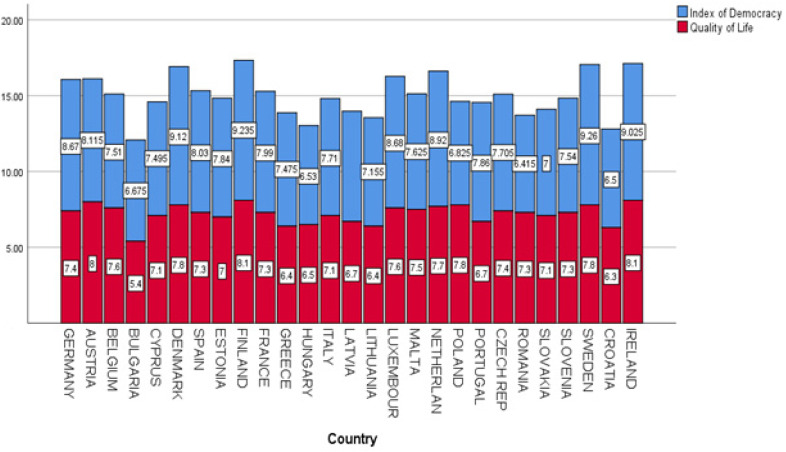
Quality of life and Democracy Index in EU-27. Average during the period 2020–2022. Sources of data: Eurostat, Quality of Life: https://ec.europa.eu/eurostat/cache/infographs/qol/index_en.html (accessed on 15 August 2022); The Economist Intelligence Unit, Index of Democracy: https://www.eiu.com/n/campaigns/democracy-index-2021/ (accessed on 6 June 2022).

We also observed significant statistical correlations between civil rights and political liberties, on the one hand, and the level of satisfaction related to the quality of life among EU-27 citizens on the other. In this respect, we estimate that there is a middle positive relation between the quality of the national democracy and the satisfaction with quality of life among EU-27 citizens, with R^2^ = 0.459 and *p* < 0.01. Figure 6 shows that countries with high Democracy Index scores are more prone to have high values related to quality of life. Finland, Austria and Ireland are the most significant countries with values > 8.00 related to the satisfaction with life among their own citizens. In contrast, Greece, Hungary, Croatia and Bulgaria are relevant cases for the lowest values registered in the field of satisfaction with quality of life. Generally speaking, we can confirm the geographical and historical cleavage between traditional democratic systems and democracies in transit after 1990. Moreover, this cleavage is met in the fields of human development, economic dimensions and political indicators.

**Figure 7:**
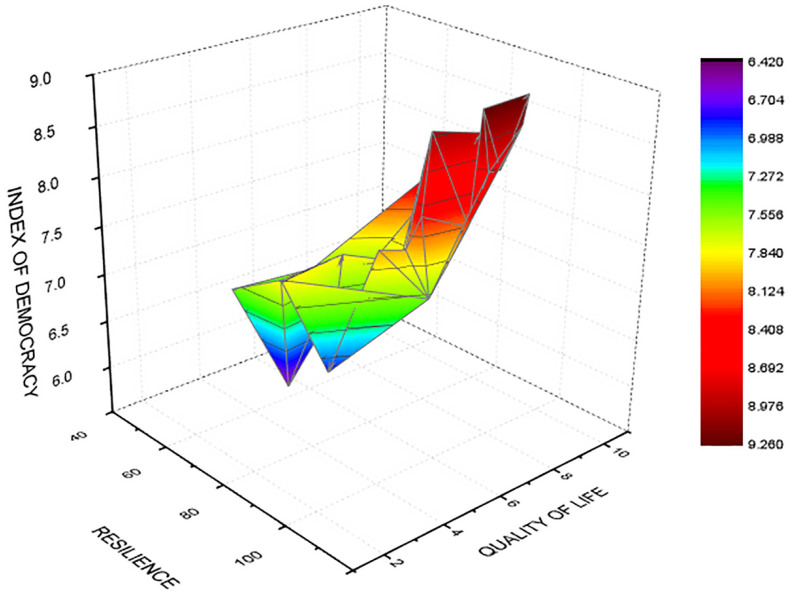
Vectors’ space for Democracy Index, Resilience and Quality of Life. Author’s quantitative determination based on statistical data presented in Section 2 (Research Methods).

An important empirical finding is related to the fact that the Resilience Index is related to democracy and satisfaction with quality of life. Figure 7 shows the interaction in a 3D space of all of these variables, suggesting that there is a significant linear association between the level of democracy, quality of life and the system capability to rebound and diminish the level of disorder. Through this result, we aim to emphasize the fact that democratic order, resilience and human development should be seen as important variables for managing the current COVID-19 crisis and further health or structural imbalances. Thus, the relation in vectors’ space between all of these variables reflects the positive association with r = 0.728, *p* = 0.01.

In conclusion, our empirical findings suggest that besides medical factors, social and political variables could be useful for a better understanding of the COVID-19 pandemic. In this respect, the quality of the healthcare systems measured by the Health Security Index could be explained through economic variables such as GDP/capita and social and political factors such as human development, resilience during health crises and the quality of national democracy. Empirical evidence shows that Scandinavian countries could be seen as emblematic case studies for understanding the interaction of medical, social, economic and political variables.

## 4. Discussion

With regard to the academic literature, this empirical study presents the relation between healthcare systems, human development, social progress, resilience, quality of life and the quality of democracy during the COVID-19 health crisis. The current pandemic generated a complex structural crisis in fields such as health systems, social dynamics, economic growth and development and political stability. For understanding both the dynamics of health security and quality of life, we use a complex model of interactions between economic, social and political variables. The key concepts related to our empirical work are represented by resilience, human development and quality of democracy. Beyond the social, economic and medical factors, we underline the idea that the political dimension could be seen as an important predictor for increasing the Health Security Index and satisfaction with quality of life for EU-27 citizens. In this respect, we argue that resilience, together with the quality of national democracy, are the main vectors involved in the absorption of medical and economic shocks during the COVID-19 pandemic [23,24,25,26]. Scholars argued that resilience represents the capacity of the social systems to rebound in conditions in which an external factor creates deep changes and disorder in the normal dynamics of social systems [53,54,71,72]. In this context, the strategy of EU political actors was to create an adequate mechanism to repair, rebound and recover in both economic and social sectors [35]. In spite of the fact that, at the political level, decision makers tried to create strategies for managing the net impact of the pandemic in the sphere of the quality of individual lives, the labor market, employability and public affairs, in practice, we can observe significant differences between the EU-27 countries in shaping their proper strategy for limiting the negative effects produced by the spread of the new coronavirus [36,37,38].

In accordance with the resilience issues, we noticed that an important impact of the COVID-19 pandemic is observed and measured in the fields of human development, social progress and quality of life. Theoretical perspectives suggest that there is a strong linear association between the evolution of the COVID-19 pandemic and the negative effects observed in the fields of human development, human security, human resources, quality of life and wellbeing. All of the social and economic effects are integrated in a model based on sustainable development. In this context, scholars emphasized the fact that human development and sustainability are affected by governmental policies for reducing the impact of the virus on society [49,50,51,52].

One of the most significant aspects of the COVID-19 pandemic is related to labor market imbalances. All of these imbalances have generated high rates of unemployment, inflation and economic decrease. Another important consequence of economic imbalances during the pandemic is reflected in the increased level of material deprivation among young people [55,56]. We can argue that governmental policies are relevant in the field of human resources, human development, education and quality of life. A decreased level of resilience and issues in the sphere of the healthcare systems management create serious damage to the perception and satisfaction related to quality of life during the COVID-19 pandemic. Despite their geographical positioning, most parts of the countries were faced with issues in managing peoples’ mental and physical health. Young people, adolescents, medical staff and teachers were negatively affected by the social isolation and lockdown measures for limiting the spreading of the respiratory infections generated by the new coronavirus.

Our research thesis is that the quality of the political regime and the rapid governmental reaction for detecting, preventing and treating the respiratory diseases caused by the SARS-CoV-2 virus could ameliorate the negative impact of the pandemic in the field of healthcare systems, human development, quality of life and wellbeing.

The statistical results show that the EU-27 is characterized by partially sustainable healthcare systems, having middle values related to the Health Security Index as statistical measure for the states’ capacity of detection, prevention and treatment of different pathogens. Values over the average of 57.32 for GHS are observed in Scandinavian countries (Finland, Denmark and Sweden), Germany and the Netherlands. The statistical results confirm that eastern European countries are characterized by partially functional healthcare systems, having GHS values of between 42.1 and 50. Our radiography of the healthcare systems and of the dynamics of the confirmed cases of COVID-19 confirms that more resilient and developed healthcare systems, with high GHS scores, are more prone to have decreased values for the total number of confirmed cases of infections and the smallest values for the COVID-19 death rate.

An important finding related to the dynamics of the healthcare systems reflects the fact that human development, social progress and economic determinants could ameliorate the negative impact of the COVID-19 pandemic in the social sphere. Moreover, the quality of the political regime could be seen as an important predictor for both the dynamics of the Global Health Security Index and the satisfaction with quality of life among the citizens of the EU-27.

Related to the research methodology guidelines, we emphasize the fact that resilience, economic factors and the quality of national democracy are relevant factors for understanding the impact of the COVID-19 pandemic in EU-27 societies. Starting from these quantitative results, we can develop models of prediction and reaction for further medical and social threats. The management of the epidemiological risks, the coordinated health policies, investment in healthcare sectors and sustainable education and an increased level of the social progress associated with a high level of resilience could represent several factors that could decrease the social and economic imbalances in EU countries. In accordance with these premises, for the research objectives O_1_ and O_2_, we can demonstrate through H_1_ that democratic political systems are more prone to develop resilient strategies for reducing the health, social and economic risks during the COVID-19 pandemic. In this respect, we measured the impact of the quality of democracy in the field of the Resilience Index. The statistical results confirm that there is a linear positive association between full democracies and more resilient political systems during the COVID-19 pandemic. Our findings are related to the theoretical perspectives reported in the academic literature [18,19,20,21,22,23,24,27,28,30,31,33,34,35,36,38,39,40,41]. For the research objective O_3_, we confirm through research the hypothesis H_2_ that social progress and human development are relevant factors for understanding and explaining the dynamics of GHS during 2020–2022. We observe that there are relevant correlations between these measures and the values associated with GHS. An increased level of social progress, as measure of sustainable development, could be a good predictor for ameliorating the statistical values of the GHS in EU-27 countries [58,60]. Both the academic literature [62,63,64,65,66,67] and empirical findings show that during the COVID-19 pandemic, human development has known negative variations. The low rates of employability, the gender gap, the increased level of socioeconomic inequality, the limited access to healthcare systems and the limited access to educational resources could be seen as several consequences of the COVID-19 pandemic in the sphere of human development [67,68,72,73,75,76,77].

In accordance with the research objective O_4,_ we argue through H_3_ that satisfaction with quality of life is related to the level of resilience of the social and political systems of the EU-27. The statistical model based on the multiple linear equation of regression shows that resilience and the quality of the political regime are predictors for satisfaction with quality of life (R^2^ = 0.785, *p* < 0.01). The academic literature underlines the fact that quality of life is strongly related to mental and physical wellbeing [76,77,78]. In this respect, scholars observed that there are differences in the perception of quality of life during the COVID-19 pandemic that depend on gender, age, education and social environment [83,84,85,86,87,88,89]. The system capacity to rebound, associated with economic and political variables, could be an important factor that explains the dynamics of the attitudes related to the perception of the quality of life. For the research objective O_5_, we confirm through H_4_ that the quality of the political regime is positively related to satisfaction with quality of life. In this respect, we determined that there is a linear statistical correlation between the Democracy Index and the quality of life in EU-27 countries during the COVID-19 pandemic (R^2^ = 0.521, *p* < 0.01). In countries such as Finland, Austria and Ireland, we estimate high values related to the quality of life. Greece, Hungary, Croatia and Bulgaria are the countries with the lowest democracy index values and decreased values related to satisfaction with quality of life.

Our approach shows that the quality of the Global Health Index (GHS) and the quality of life in the EU-27 depend on political, economic and social variables. In this respect, for full democracies, which are prevalent in Scandinavia, the Democracy Index and the GDP/capita are seen as important factors related to the quality of health security. In contrast, in flawed democracies, we could not estimate a significant positive statistical correlation. Moreover, in all of the political systems of the EU-27 countries, we estimate that the social progress index (as measure for sustainable development) and the human development index are factors related to middle positive coefficients of correlation with the GHSI. Beyond the healthcare dimension, our paper stresses that it is important to emphasize the role played by the satisfaction related to quality of life and wellbeing during the COVID-19 pandemic. In this respect, analytical models suggest that there are two significant factors related to satisfaction with quality of life: resilience and the quality of the democracy. Starting here, we emphasize the role played by resilience and political factors in managing further epidemics or pandemics in the EU-27. A coordinated mechanism for health policies together with an increased level of detection, prevention and treatment of infectious diseases could be associated with the quality of democracy, resilience and social progress for having an optimal response in the face of different medical, social and economic crises.

## 5. Conclusions

In conclusion, the main goal of the paper is to link medical, social and economic variables to the quality of the political regimes across the EU-27 countries during the COVID-19 pandemic. Our research shows the magnitude of the COVID-19 pandemic in the field of the Health Security Index and quality of life and wellbeing. In this context, our empirical findings show that the EU-27 have a middle value for the Global Health Security Index (GHSI), which reflects a limited capability to prevent and respond to different health threats such as pandemics or epidemics. This limited capacity to detect, prevent and react during pandemic times is associated with several social, economic and political variables, such as social progress index, human development, GDP/capita and Democracy Index. An increased value for GHS could be seen as an optimal mechanism for the early detection of further pathogens with a moderate or severe impact on the social–economic sphere. In this respect, the EU-27 can use GHS as an “early warning unit” in accordance with political and social indicators. Our statistical results confirm, in accordance with the theoretical premise, that full democracies are more prone to develop qualitative healthcare systems with high scores associated with GHS. Scandinavian countries could be seen as emblematic states, in which a high level of democracy is strongly correlated with a high degree of GHS. Moreover, the Resilience Index predicts both the evolution of the healthcare systems and the satisfaction related to quality of life and wellbeing during the COVID-19 pandemic. In addition to social progress and human development, this article aims to emphasize the fact that the key variables for explaining the evolution of the COVID-19 pandemic and the quality of life of the EU-27 citizens during this crisis are represented by the Democracy Index and the Resilience Index. Democracy and resilience could be seen as important predictors in crucial moments characterized by different types of medical or socioeconomic risks and threats. The empirical findings might be considered useful for those involved in elaborating a theoretical approach addressing the issue of healthcare systems and quality of life and for decision makers interested in shaping health policies for reducing the pressure and the impact of the COVID-19 virus on society. This study has several limits imposed by the limited data collection and the short period of time in which scholars had the opportunity to analyze the phenomenon in the academic literature. The same thing could be noted for the theoretical perspectives related to the further impact of COVID-19 in social and healthcare fields. Our future research directions will be oriented towards a better understanding of the COVID-19 pandemic’s impact on the social, economic and medical fields in a comparative approach between western and eastern European countries.

## Figures and Tables

**Table 1:** Research variables.

Variable	Symbol	Unit of Measurement	Data Source
Index of Democracy	ID	[0–10]	Economist Intelligence Unit [101]
Human Development Index	HDI	[0–1]	United Nations Development Programme [58]
Quality of Life/Life Satisfaction	QL	[0–10]	Eurostat [77] OECD Better Life Index [76]
Global Health Security Index	GHS	[0–100]	Johns Hopkins Center for Health Security [38]
GDP/Capita	GDP	$/capita	World Bank [102]
Civil Liberties	CL	[0–10]	Economist Intelligence Unit [101]
Resilience Index	RI	[0–100]	FM Global [103]
Social Progress Index	SPI	[0–100]	Social Progress Imperative [100]
Confirmed COVID-19 cases (2020–2022)	Cov-19	[0–n]	Johns Hopkins Coronavirus Resource Center [104]
COVID-19 Death Rate (2020–2022)	DRC	[0–100}	Johns Hopkins Coronavirus Resource Center [104]
Proportion of Population Fully Vaccinated	VR	[0–100]	Johns Hopkins Coronavirus Resource Center [104]

**Table 2:** Descriptive statistics for research variables. Average of the values 2020–2022 ^1^.

		Global Health Security Index	Index of Democracy	Human Development Index	Quality of Life	Resilience Index	Social Progress Index
Minimum		39.75	6.42	0.82	3.00	60.80	78.41
Maximum		71.45	9.26	0.96	10.00	100.00	92.26
Percentiles	25	53.33	7.18	0.86	5.10	68.60	83.86
	50	56.92	7.70	0.89	6.10	81.20	86.58
	75	64.76	8.58	0.93	8.10	92.35	89.26
Range		31.70	2.85	0.14	7.00	39.20	13.85
Mean		57.32	7.80	0.89	6.23	80.66	86.42
Median		56.92	7.70	0.89	6.10	81.20	86.58
Std. Deviation		7.92	0.85	0.03	1.90	12.06	3.70
Variance		62.85	0.73	0.01	3.63	145.52	13.72

^1^ Sources of data: Global Health Security, 2021: https://www.ghsindex.org/about/ (accessed on 10 July 2022); Economist Intelligence Unit, 2020: https://pages.eiu.com/rs/753-RIQ-438/images/democracy-index-2020.pdf (accessed on 6 June 2022), 2021: https://www.idea.int/gsod/sites/default/files/2021-11/the-global-state-of-democracy-2021_0.pdf (accessed on 5 June 2022); United Nations Development Programme: Human Development Report: https://hdr.undp.org/reports-and-publications (accessed on 15 July 2022); OECD Better Life Index: https://www.oecdbetterlifeindex.org/countries/austria/ (accessed on 25 July 2022); Eurostat, Quality of Life: https://ec.europa.eu/eurostat/cache/infographs/qol/index_en.html (accessed on 15 August 2022); FM Global Resilience Index: https://www.fmglobal.com/ (accessed on 25 July 2022); Social Progress Imperative, Social Progress Index: https://www.socialprogress.org/europe (accessed on 20 August 2022).

**Table 3:** Multiple linear equation of regression: Global Health Security Index in full democracies ^1^.

Model		Unstandardized Coefficients		Standardized Coefficients	t	Sig.	Collinearity Statistics	
		B	Std. Error	Beta			Tolerance	VIF
1	(Constant)	74.622	3.544		21.058	0.000		
	GDP per capita	0.000	0.000	−0.787	−3.828	0.004	1.000	1.000
2	(Constant)	15.825	13.357		1.185	0.270		
	GDP per capita	0.000	0.000	−0.955	−7.773	0.000	0.905	1.104
	Democracy Index	7.095	1.593	0.547	4.453	0.002	0.905	1.104

^1^ Author’s quantitative model based on statistical data presented in Section 2 (Research Methods).

**Table 4:** Multiple linear equation of regression: predictors for quality of life ^1^.

Model		Unstandardized Coefficients		Standardized Coefficients	t	Sig.	Collinearity Statistics	
		B	Std. Error	Beta			Tolerance	VIF
1	(Constant)	−9.435	2.392		−3.944	0.001		
	Democracy Index	1.961	0.298	0.821	6.583	0.000	1.000	1.000
2	(Constant)	−9.641	2.138		−4.508	0.000		
	Democracy Index	1.245	0.390	0.521	3.193	0.005	0.466	2.146
	Resilience Index	0.071	0.028	0.410	2.515	0.021	0.466	2.146

^1^ Author’s quantitative model based on statistical data presented in Section 2 (Research Methods).

## Data Availability

The quantitative data were extracted and collected from secondary sources and archives as follows: Johns Hopkins Center for Health Security, Global Health Security, 2021: https://www.ghsindex.org/about/ (accessed on 10 July 2022). The Economist Intelligence Unit, 2020: https://pages.eiu.com/rs/753-RIQ-438/images/democracy-index-2020.pdf (accessed on 6 June 2022); https://www.idea.int/gsod/sites/default/files/2021-11/the-global-state-of-democracy-2021_0.pdf (accessed on 5 June 2022); United Nations Development Programme, Human Development Report: https://hdr.undp.org/reports-and-publications (accessed on 15 July 2022); OECD, OECD Better Life Index: https://www.oecdbetterlifeindex.org/countries/austria/ (accessed on 25 July 2022); FM Global Resilience Index: https://www.fmglobal.com/ (accessed on 25 July 2022); Social Progress Imperative, Social Progress Index: https://www.socialprogress.org/europe (accessed on 20 August 2022); Eurostat, Quality of Life: https://ec.europa.eu/eurostat/cache/infographs/qol/index_en.html (accessed on 15 August 2022); World Bank, World Bank Data: GDP 2020-2021: https://data.worldbank.org/indicator/NY.GDP.MKTP.CD (accessed on 16 August 2022); Johns Hopkins Coronavirus Resource Center, World Countries: https://coronavirus.jhu.edu/region (accessed on 20 September 2022).
